# CBR-based integration of a hydrodynamic and water quality model and GIS—a case study of Chaohu City

**DOI:** 10.1007/s11356-018-3862-5

**Published:** 2019-01-08

**Authors:** Zhenliang Liao, Can Zhou, Wenchong Tian, Tiantian Hu, Ru Guo

**Affiliations:** 1 School of Civil Engineering, Xinjiang University, Xinjiang, 830046 People’s Republic of China; 2 State Key Laboratory of Pollution Control and Resource Reuse, UNEP-Tongji Institute of Environment for Sustainable Development, College of Environmental Science and Engineering, Tongji University, Shanghai, 200092 People’s Republic of China; 3 Shanghai Institute of Pollution Control and Ecological Security, Shanghai, 200092 People’s Republic of China; 4 Key Laboratory of Yangtze River Water Environment (Ministry of Education), Tongji University, Shanghai, 200092 People’s Republic of China

**Keywords:** Hydrodynamic, Water quality model, GIS, Case-based reasoning, Decision support system, Case retrieval

## Abstract

Monitoring on urban water environment and analysis of engineering improvement measures are intricate and time-consuming tasks. In previous studies, the integration of hydrodynamic and water quality models and geographical information system (GIS) usually takes three approaches: loose coupling, tight coupling, and full coupling. However, this paper adopted a special loose coupling approach—case-based reasoning (CBR) to develop an integrated decision support system. This was characterized by invoking the case base stored in the GIS platform as the output of the model. The fused capability of model’s water quality predication and strong spatial data processing analysis of GIS can be realized at the same time by integration. The functionality of the integrated system was illustrated through a case study of Chaohu, a medium-sized city in China, which includes case retrieval, result interpretation, and the visual display in the GIS platform. Results verified the feasibility and operability of the developed method. As a useful tool, the integrated decision support system makes it simpler and more convenient for decision makers to make decisions efficiently and quickly.

## Introduction

With the accelerated urbanization progress, water environmental problems have become increasingly prominent. In order to understand the actual situation of water environment, a more suitable method is required to simulate and predict. Mathematical models of water environment have a strong ability to calculate and predict complex water environment (Bermúdez and García-García 2012). However, such complex models generally need a large number of geographically referenced information, and thus require much efforts in preparing input data as well as displaying output results. On the other hand, water environment changes with the complex space and region, and thus, it is inseparable from spatial geographic information. Although geographical information system (GIS) has a powerful capability of geographic information analysis and visualization, it cannot simulate and predict the water environment. Therefore, how to achieve the complementary advantages of the two is worth studying.

Great efforts have been made over the past years to integrate the hydrodynamic and water quality model and GIS to build a comprehensive decision-making water environment management system (Fernandes et al. 2014; Li 2006; Liu 2005; Zhang 2012; Choi et al. 2005). Such integrations not only make up for the weakness of the calculation and prediction of GIS but also make the simulation results spatially visualized and intelligently analyzed. Previous scholars have mainly summarized approaches of the integration of hydrodynamic and water quality models and GIS into three approaches: loose coupling, tight coupling, and full coupling. The GIS functions both as a pre-processor and a post-processor to the modeling system by means of loose coupling. Tight coupling is the integration of the models and GIS under a common interface. In full coupling, a GIS can be added to the modeling system or the models are developed within the GIS environment (Huang and Jiang 2002; Vairavamoorthy et al. 2007). For example, Ng et al. (2009) made a research on the integration of GIS with a complex three-dimensional hydrodynamic sediment and heavy metal transport numerical model. Jia (2001) made an integration of GIS with the surface water quality model WASP5 based on a case study of Miyun Reservoir, using a tight coupling method. Huang et al. (2013) integrated GIS with the dynamic water quality model on the basis of a full coupling system, which realized the dynamic visual display and efficient prediction of sudden water pollution accidents in a relatively short time.

The loose coupling approach has a strong feasibility of system development and an easy application, but it has a tedious system of data exchange without a uniform interface. The tight coupling approach avoids the frequent file exchanges between two systems, but it is unable to realize the interaction between users. Full coupling can realize the thorough integration and data sharing of model and GIS on the same user interface. However, too much effort and time are required to develop the system and ordinary people except technical developers cannot compile, which is not applicable for the integration of non-open source commercial software.

At present, for small- and medium-sized cities in China, due to the lack of managers with a certain amount of professional knowledge and limited investment, it is not easy for them to build a tight and full coupling system with high manpower and financial costs. What it needs is an easy-to-use operating system with fewer requirements for operators. Tight and full coupling approaches studied by previous scholars are costly and require the user to have certain professional background knowledge. Although loose coupling has a relatively low precision, it has a simpler operation interface and fewer professional knowledge requirements, so it is more suitable for small- and medium-sized cities.

Based on the loose coupling method, this paper introduces another approach, that is, case-based reasoning (CBR). A new problem can be solved by searching for a most similar case in case base and reusing it in the new problem situation (Aamodt and Plaza 1994). This method avoids a large number of model calculations, which simplifies user operation and does not require users to have a high level of professionalism. It is distinct from the other general loose coupling methods because cases generated by the model can be stored in the GIS to form the case base. A new problem can be solved by using CBR to retrieve and invoke the most similar case, so there is no intermediate file generation and the manager can perform visual display of results in the unified GIS platform.

Various kinds of CBR systems with different reasoning mechanisms have been developed since 1980s (Aamodt and Plaza 1994; Kolodner 1993). During the past decade, CBR and its relevant techniques have been applied in many ways, including product design (Kwong and Tam 2002), disease diagnosis (Saraiva et al. 2016), fault diagnosis (Yang et al. 2004), intelligent control (Yan et al. 2012), and numerical prediction (Jalali and Leake 2016). In addition, many experts and scholars use the model to build a case base and then use CBR to solve practical problems. For example, Jiang (2007) built a case base by MIKE21, forming a quickly checked manual for oil spill preparedness of Huangpu River to solve the sudden oil spill pollution in Huangpu River. Liao et al. (2005) developed a model to simulate major urban accidents and respectively constructed emergency preparedness bases for different functional areas in cities and different types of accidents. Sørensen et al. (2007) integrated the Danish emergency response model of the atmosphere (DERMA) with the Accident Reporting and Guidance Operational System (ARGOS) nuclear decision support system mainly for nuclear emergency preparedness purposes. However, these studies mainly focused on sudden environmental pollution and paid less attention on analysis of routine engineering plans. Compared with that, this paper proposes an integration method used for the daily monitoring on urban water environment and engineering analysis. This approach makes it more convenient and efficient for managers to operate the system to make decisions quickly.

The primary objective of the paper is to adopt the CBR method to make a decision support system based on the integration of a hydrodynamic and water quality model and GIS. The detailed objectives were to (1) establish a hydrodynamic and water quality model in a medium-sized city called Chaohu, southeast China, with specific function demanding, case designing, and case reasoning steps; (2) make pre-generated cases of the model stored in GIS, which forms the case base; and (3) directly call the case from the case base by CBR method as the output of the current model result.

## Methodology

### Study area

In this study, a medium-sized city called Chaohu was taken as an example to develop a CBR-based decision support system which integrated a hydrodynamic and water quality model and GIS. Chaohu City is located in Anhui Province, China, near the Yangtze River, surrounded by abundant clean water resources.

There are mainly two problems in the study area. Firstly, due to the lack of internal circulation and the lack of external clean water injection, the self-purification ability of the water body is relatively weak. Secondly, many sewage outlets along the river channel have not been intercepted, which poses a great threat to the safety of water environment. Therefore, two kinds of improvement measures are made including water diversion and sewage interception.

In order to have a quicker and more intuitive understanding of the performance of improvement measures, the hydrodynamic and water quality model and GIS can be integrated. But how to integrate them in a better way is worth studying. Considering that the investment in Chao Lake is limited and the manager lacks certain professional knowledge, it is suitable to use CBR method to integrate, which is simpler and more convenient with lower operation threshold.

### Framework of the integrated decision support system

**Fig. 1:**
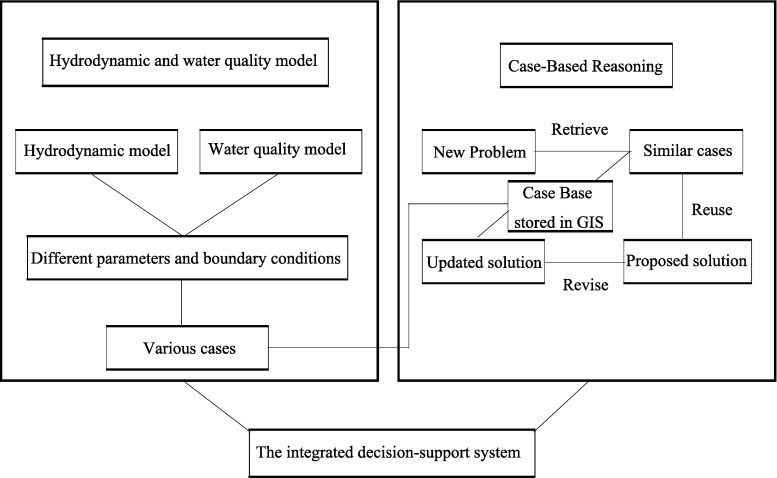
The framework of the decision support system

Typically, the most commonly used model of CBR includes four steps: case retrieval, case reuse, case revision, and case retention (Aamodt and Plaza 1994). Framework of the integrated decision support system is shown in Fig. 1.

**Fig. 2:**
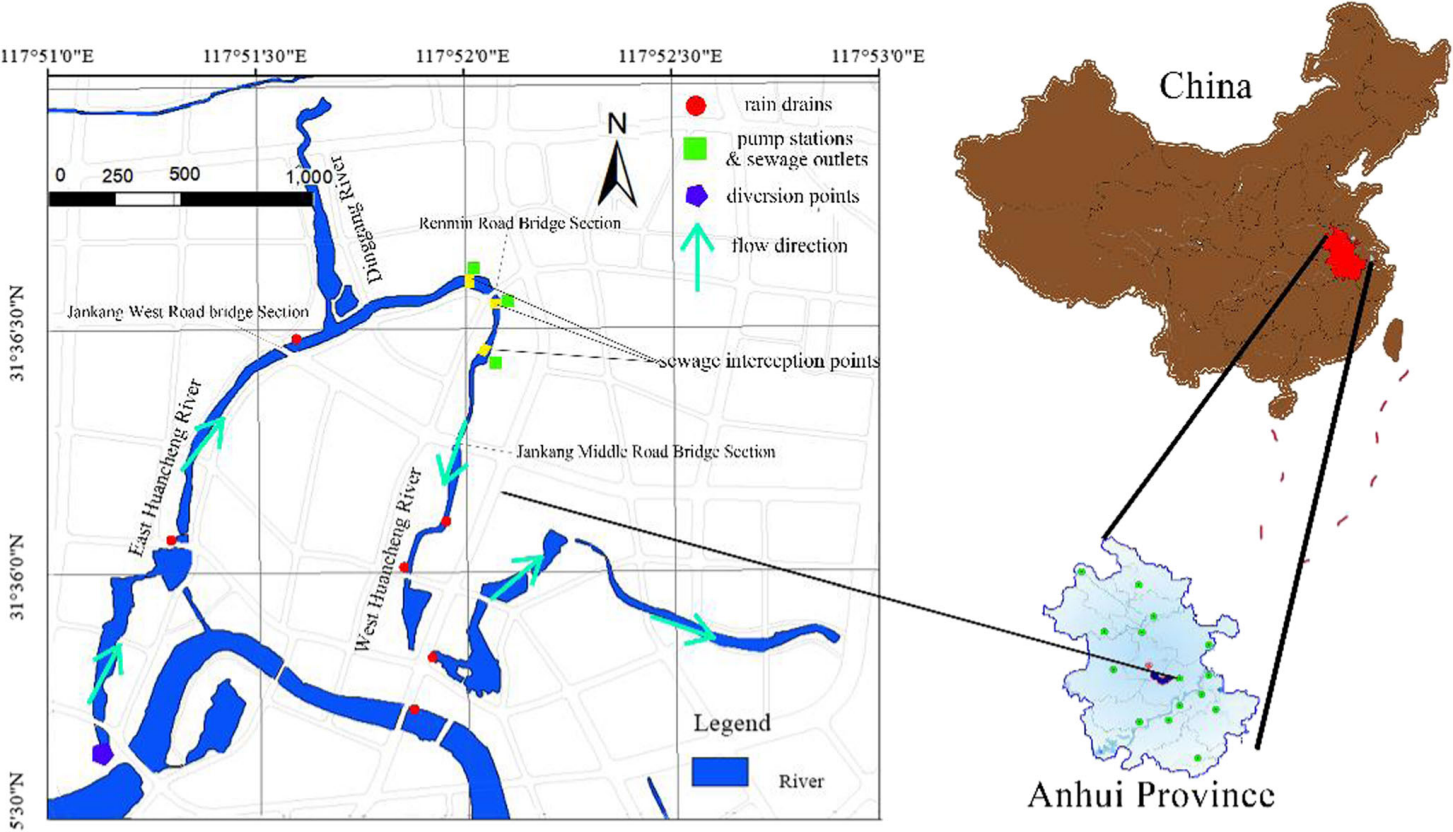
Map of the regional water system distribution

Select the appropriate hydrodynamic and water quality model in advance to design cases under various working conditions and then save them as the case base. When a new problem arises, the most similar case can be retrieved by CBR and displayed in the GIS platform. Distribution of the river network in this study area is shown in Fig. 2.

### Hydrodynamic and water quality model

Since the longitudinal length of Chaohu is much larger than its width and depth, its river network can be regarded as a one-dimensional flow. With the widespread use of MIKE11 and its targeting at river networks of small- and medium-sized inland cities, the modeling software MIKE11 was employed for water diversion and sewage interception simulation (Tompson et al. 2004). The theoretical basis of its hydrodynamic module is the Saint-Venant equations, and the basis of the water quality module is the one-dimensional convection diffusion equation.

Saint-Venant equations were applied to describe the water flow, which include mass and momentum conservation, as follows (Doulgeris et al. 2012):

1 $$ \frac{\partial A}{\partial t}+\frac{\partial Q}{\partial x}=\mathrm{q} $$

2 $$ \frac{\partial Q}{\partial t}+\frac{\partial }{\partial x}\left(\frac{\alpha {Q}^2}{A}\right)+ gA\frac{\partial h}{\partial x}+\frac{n^2 gQ\left|Q\right|}{A{R}^{\frac{4}{3}}}=0 $$

where *Q* is the discharge (m^3^/s), *A* is the cross-sectional area of the flow (m^2^), *q* is the lateral inflow or outflow (m^2^/s), *h* is the water surface elevation (m), *n* is the manning resistance coefficient, *R* is the hydraulic radius (m), *α* is the correction factor (dimensionless), *g* is the acceleration due to gravity (m/s^2^), and *x* and *t* are the longitudinal distance (m) and time (s), respectively.

In terms of a one-dimensional flow, the advection dispersion equation for pollutant transportation can be formulated as (Xue et al. 2015):

3 $$ \frac{\partial (AC)}{\partial t}+\frac{\partial (QC)}{\partial x}=\frac{\partial }{\partial x}\left({AE}_x\frac{\partial C}{\partial x}\right)- KC+{W}_p $$

where *C* is the cross-sectional average solute or suspended sediment concentration (mg/L), *E*_*x*_ is the sectional average longitudinal dispersion coefficient (m^2^/s), *K* is the first-order effective decay rate (/day) and *W*_*p*_ is the sectional average source or sink term (kg/(s m^3^)), which includes the external point source load and the nonpoint source load. Detailed information is shown in the Appendix.

### Case-based reasoning

#### Case design

MIKE11 was employed for water diversion and sewage interception simulation. Different model parameters and boundary conditions constitute various kinds of engineering plans which were then stored in the decision support system to form the case base. According to the actual situation of the study area, besides the factor like rainfall runoff which affects water quality, engineering plans could be divided into three categories: water diversion, sewage interception, and the combination of water diversion and sewage interception. Therefore, model variables were water temperature, rainfall, diverted water flow and diverting time, and interception rate.

Based on the local measured water temperature, 4 °C, 7.5 °C, 11 °C, 14.5 °C, 18 °C, 21.5 °C, and 25 °C were chosen as the model parameters. According to the Pilgrim and Cordery method (Jiang 2015), three types of precipitations were designed whose rainfall were over 20 mm, 10~20 mm, below 10 mm, respectively. Another modeling software MIKE URBAN (DHI, www.mikepoweredbydhi.com) was used to simulate six rain drains of the East and West Huancheng River and three combined pumping stations under the three precipitation conditions, respectively. After that, results can be input into MIKE11 as boundary conditions to calculate simulation results. Diverted water flows were 1.5 m^3^/s, 3 m^3^/s, and 5 m^3^/s and diverting time were 15 h, 20 h, and 30 h while interception rates were 30%, 80%, and 100%. Reasons for chosen model parameters are briefly as follows:

1. Water was diverted from the upstream of West Huancheng River. Pollutant removal rate per unit diverted water flow (PRUWF) increased at the beginning of water diversion and subsequently decreased with the increase of diverted water flow. When the diverted water flow was 5 m^3^/s, PRUWF had the maximum value indicating the highest efficiency of the water quality improvement. At the same time, when the total amount of water diversion was the same, water quality could be better improved with the decreased diverted flow. Therefore, with a certain gradient, diverted water flows of 1.5 m^3^/s, 3 m^3^/s, and 5 m^3^/s were chosen.
2. In order to realize the improvement of East and West Huancheng River, the optimal clean water diversion was operated under the condition of a flow of 5 m^3^/s for 48 h and the total amount of water was 864,000 m^3^. Therefore, several time values (5 h, 10 h, 15 h, 20 h, 25 h, 30 h) lower than 48 h were selected from the perspective of economy. However, it is not necessary to store a case if the water improvement is not significant, like the cases with diverting time lower than 15 h (Gu et al. 2017). Thus, with a certain gradient, diverting time of 15 h, 20 h, and 30 h were chosen.
3. In order to meet the environmental quality standard for surface water of local requirement, different sewage interception rates were simulated (30%, 50%, 80%, 100%). In terms of methodology, each simulated value can be selected as a case which can more comprehensively reveal the actual situation of the region. For each case, however, the process of case design, case calculation, and case saving was needed. Thus, in terms of the typicality of cases, interception rates were selected at a certain gradient of 30%, 80%, and 100% to build the case base first and try to operate the method proposed in this paper, which highlights the research idea and method of this paper.

More details of the parameters can be found in the previous research (Gu et al. 2017).

#### Case-based reasoning

Case applied in CBR is a piece of knowledge with contextual information and empirical expression to solve the accident. In practical applications, it needs to be converted into a computer-operable data format (Zhang and Liu 2002; Lei et al. 2006; Maher and Garza 1995). Case structure is generally represented as case = (problem description, solution, result assessment), while the part of result assessment may not necessarily appear in some systems (Li et al. 2009). Common representing methods of cases are production, framework, semantic web, decision tree, object-oriented, etc. This paper uses the framework method.

## Case retrieve

At present, there are three following commonly used retrieval methods: nearest neighbor approach, inductive reasoning approach, and knowledge-based indexing approach (Wang 2009). Nearest neighbor approach was employed in this study, and the similarity function is used as follows:

4 $$ \mathrm{Sim}\left(\mathrm{T},\mathrm{S}\right)=\frac{\sum_{\mathrm{i}=1}^{\mathrm{n}}\mathrm{f}\left({\mathrm{T}}_{\mathrm{i}},{\mathrm{S}}_{\mathrm{i}}\right)\times {\upomega}_{\mathrm{i}}}{\sum_{\mathrm{i}=1}^{\mathrm{n}}{\upomega}_{\mathrm{i}}} $$

where *T* is the target problem, *S* is the retrieved case, *n* is the total number of case attributes, *i* is the specific attribute number from 1 to *n*, *f* is the similarity function of the attribute *i* of *T*, and *S*, *ω* is the importance weight of the attribute *i*.

Heterogeneous Euclidean-overlap metric (HEOM) approach was employed to confirm the similarity function of retrieved attributes (Huang 2009; Li and Li 2011). Attribute hierarchical model (AHM) was applied to calculate the weight of each attribute (Wang 1990). Firstly, the sensitivity analysis of each model parameter was needed. However, since the sensitivity of both case type and rainfall cannot be quantitatively calculated, they were determined by a large number of case data and compared with each other. Secondly, according to the AHM attribute scoring standard table, relative importance of each attribute was obtained by the calculated sensitivity of each parameter, and then, the weight conversion formula was introduced to get the weight of the six retrieved attributes respectively. Finally, total similarity of each case can be obtained by means of Eq. (4).

## Case reuse

Case reuse refers to the application of the case when the most similar case has been retrieved. In terms of the content of the reused case, case reuse can be mainly divided into two types: result multiplexing and method multiplexing (Marir 1994; Shi 2002; Zhou 2005). Result reuse means that when solution of the retrieved case needs to be adjusted, it is converted into the corresponding solution in the new case by using some conversion rules while method reuse pays more attention to the solving method of the retrieved case rather than the result. The specific application depends on the actual situation.

## Case revise

Case revise generally refers to the proper modification and adjustment of the retrieved case solution, using the rule-based rewriting method so as to meet the requirements of the current problem. The theoretical basis of the water environmental model is needed in case revise to establish the relationship between the attribute of case and the result of simulation. Previous studies have summarized some qualitative rules. For example, Gu et al. (2017) found that an increase of both diverted water flow and diverting time improves water quality and broadens the purifying scope. However, simulation results were presented in a two-dimensional table format and it was impossible to change the tabular data quantitatively. What is more, various kinds of data in the two-dimensional table have their own specific change pattern which may not the same. If the same rule is used to modify the result, it will cause a great error. Therefore, this study does not temporarily perform case revise and leaves the modification of the case to the user. If the current case has a very low similarity to previous cases in case base (below the similarity threshold), the current case will be recorded and simulated separately by using the model, and the new case will be stored in case base afterwards.

## Case retain

Case retain refers to the continuous preservation of new cases and their respective solutions, reflecting the self-learning process of CBR. But unconditionally saving the case will reduce the operating efficiency of the system and prolong the retrieval time. Therefore, the similarity threshold is needed and cases with too low or too high similarity will not be preserved in actual applications. Specific threshold depends on the targeted accuracy. In this study, the upper similarity threshold is up to 0.9 and the lower one is 0.3. When the new case has a similarity between 0.3 and 0.9, it will be automatically saved in case base.

### Development of GIS

Methods or tools adopted in the secondary development of GIS were selected (Table 1). Through the secondary development of GIS, elements like river network, drainage system, pollution source, etc. were integrated into the coupled decision support system. When a new problem occurs, the most similar case will be retrieved and two-dimensional tables and change curves of water level, water flow, and water quality can be displayed in the GIS platform. Besides that, programming can also be used to combine the visual results of the most similar case with the map roaming function of ArcGIS, that is, the number of channel sections on the GIS map matches that in case base. If a certain channel section on the GIS map is chosen, its process of the changing water level, water flow, and water quality over time will appear respectively.

**Table 1:** The secondary development of GIS

Development items		Methods or tools
Software environment	The component package of secondary development	Component-based secondary development-ArcGIS Engine
	Development environment	Visual Studio.NET
	Selection of the database	Microsoft Access
	The way of GIS to access the database	Object Linking and Embedding Database (OLE DB)
Hardware environment	CPU model	Intel®Core™i7-4700
	CPU operation frequency	2.40 GHz
	Memory capacity	8.00 G

## Results

### Case base

#### Case design

Variables selected in “Case design” were combined to construct cases and the case base of the water environmental decision support system was initially formed. Take water temperature as a control variable, when water temperature was 4 °C, firstly, three types of precipitations were designed for rainy days. Secondly, engineering plans for dry days could be divided into three scenarios: water diversion, sewage interception, and both water diversion and sewage interception. For water diversion, three kinds of both diverted water flow and diverting time were combined respectively, which constituted nine cases in total. For sewage interception, interception rate of 30%, 80%, and 100% were selected, which formed three cases. For both water diversion and sewage interception, cases are shown in Table 2 and the number of total cases was 42. In consideration that seven values of temperature were chosen, the total number of cases of all temperatures was 294.

**Table 2:** Designed cases at 4 ^°^C for both water diversion and sewage interception

Diverted water flow	Diverting time	Interception rate
1.5 m^3^/s	15 h	30%
		80%
		100%
	20 h	30%
		80%
		100%
	30 h	30%
		80%
		100%
3 m^3^/s	15 h	30%
		80%
		100%
	20 h	30%
		80%
		100%
	30 h	30%
		80%
		100%
5 m^3^/s	15 h	30%
		80%
		100%
	20 h	30%
		80%
		100%
	30 h	30%
		80%
		100%

#### Development of the case base

**Fig. 3:**
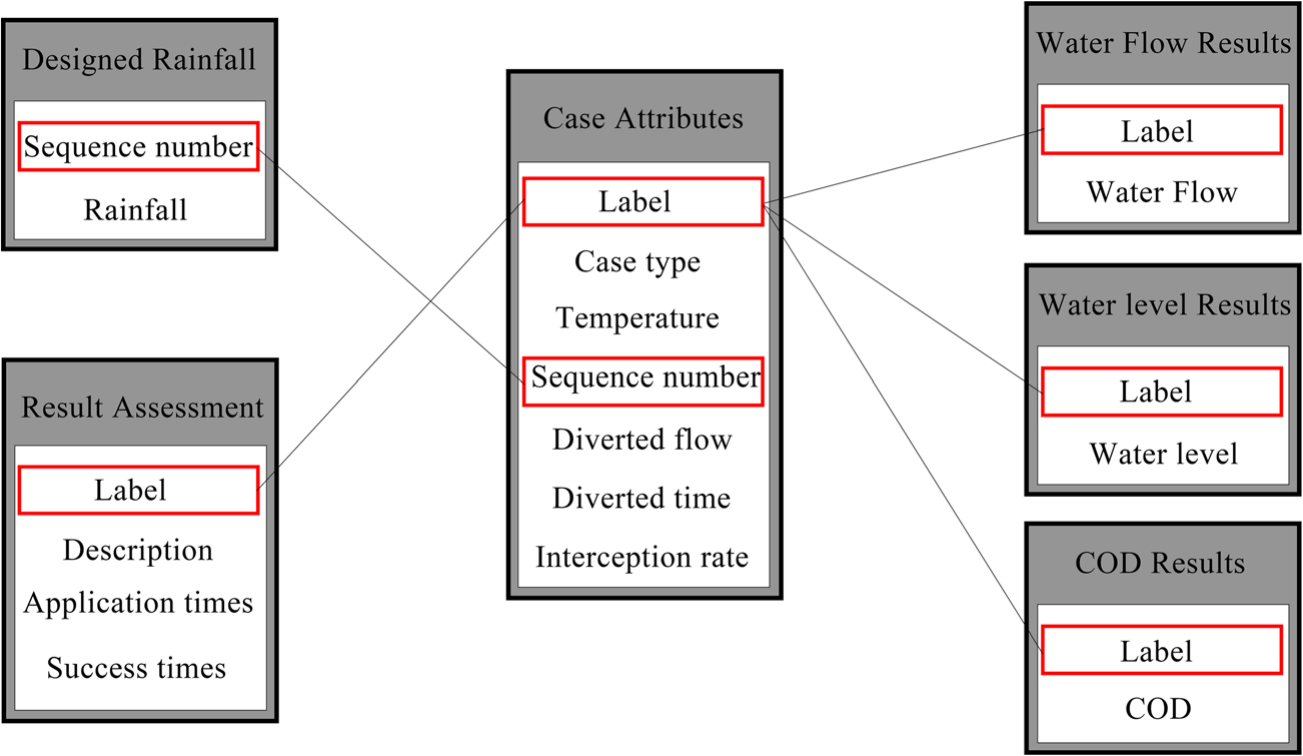
Connection mode between all data tables

Three aspects of information were mainly included in the case base of the Access database: attributes, simulated results, and assessment of simulated results. Therefore, eight tables were contained in the Access database: tables of case attributes, designed rainfall sequences, water level, water flow, water quality (COD, NH4^+^-N, TP) and result assessment, respectively. Take COD as an example of water quality result, tables were connected by the case code label (Fig. 3).

### Case-based reasoning

Firstly, according to the method introduced in “Case-based reasoning,” sensitivity values of parameters like water temperature, diverted water flow, diverting time, and interception rate were calculated (Table 3). Secondly, case type was summarized by the practical application. For example, the actual situation of Chaohu City determines that the local case types can be mainly divided into four categories: rainfall runoff, water diversion, sewage interception, and the combination of water diversion and sewage interception. If the condition changes during the actual engineering plans, other case type like regulation and storage can be added. Case type reflects the local actual working condition, which is the direct factor determining model boundary conditions, while different boundary conditions is one of the main reasons determining the model simulation results. Therefore, case type is the prerequisite for all retrieval attributes and its relative importance is the highest among the six retrieval attributes. Thirdly, sensitivity of rainfall was achieved by being compared with sensitivity of water temperature. Assuming that the water temperature was 14.5 °C, water flow was 3 m^3^/s, water diversion was 20 h, and interception rate was 30%; influence of three kinds of precipitations on retrieved case was studied (Table 4). Results show that retrieved label was the same if only rainfall type was changed, which indicates that the most similar case retrieved was the same. Next, assuming that the rainfall type was 1, water flow was 3 m^3^/s, water diversion was 20 h, and interception rate was 30%; influence of seven temperatures on retrieved case was studied (Table 5). Results show that if only water temperature was changed, different labels were retrieved, which indicates that different water temperatures have a certain impact on the retrieved case.

**Table 3:** Sensitivity of attributes

	Value	COD	|S|
Water temperature	14.4 °C	21.954	0.236
	16.2 °C	21.943	
	18 °C	21.932	
	19.8 °C	21.917	
	21.6 °C	21.9	
Diverted water flow	2.4 m^3^/s	45.217	1.115
	2.7 m^3^/s	45.08	
	3 m^3^/s	44.953	
	3.3 m^3^/s	42.197	
	3.6 m^3^/s	39.694	
Diverting time	16 h	34.321	0.889
	18 h	34.718	
	20 h	33.975	
	22 h	33.534	
	24 h	33.287	
Interception rate	0.56	37.774	0.671
	0.63	37.750	
	0.7	37.702	
	0.77	37.681	
	0.84	37.667

**Table 4:** Influence of rainfall on retrieved case

Rainfall id	Label	Similarity
1	117	0.91496
2	117	0.91581
3	117	0.93265

**Table 5:** Influence of water temperature on retrieved case

Water temperature	Label
4 ^°^C	117
7.5 ^°^C	144
11 ^°^C	171
14.5 ^°^C	198
18 ^°^C	225
21.5 ^°^C	252
25 ^°^C	279

Therefore, the influence of rainfall on the retrieved case is less than water temperature. Based on the calculated sensitivity values of other four attributes, sensitivity values of the six attributes in sequence were as follows: case type>diverted water flow>diverting time>interception rate>water temperature>rainfall. The relative importance and weights of all the retrieval attributes are shown in Table 6 and Table 7, respectively.

**Table 6:** Relative importance of all the retrieval attributes

Retrieval attribute	Case type	Diverted water flow	Diverting time	Interception rate	Water temperature	Rainfall
Case type	1	2	3	4	5	6
Diverted water flow	1/2	1	2	3	4	5
Diverting time	1/3	1/2	1	2	3	4
Interception rate	1/4	1/3	1/2	1	2	3
Water temperature	1/5	1/4	1/3	1/2	1	2
Rainfall	1/6	1/5	1/4	1/3	1/2	1

**Table 7:** Weights of all the retrieval attributes

Retrieval attribute	Rainfall	Water temperature	Case type	Diverted water flow	Diverting time	Interception rate
Weight	0.0415	0.0897	0.2919	0.2437	0.1926	0.1407

### Construction of the GIS platform

**Fig. 4:**
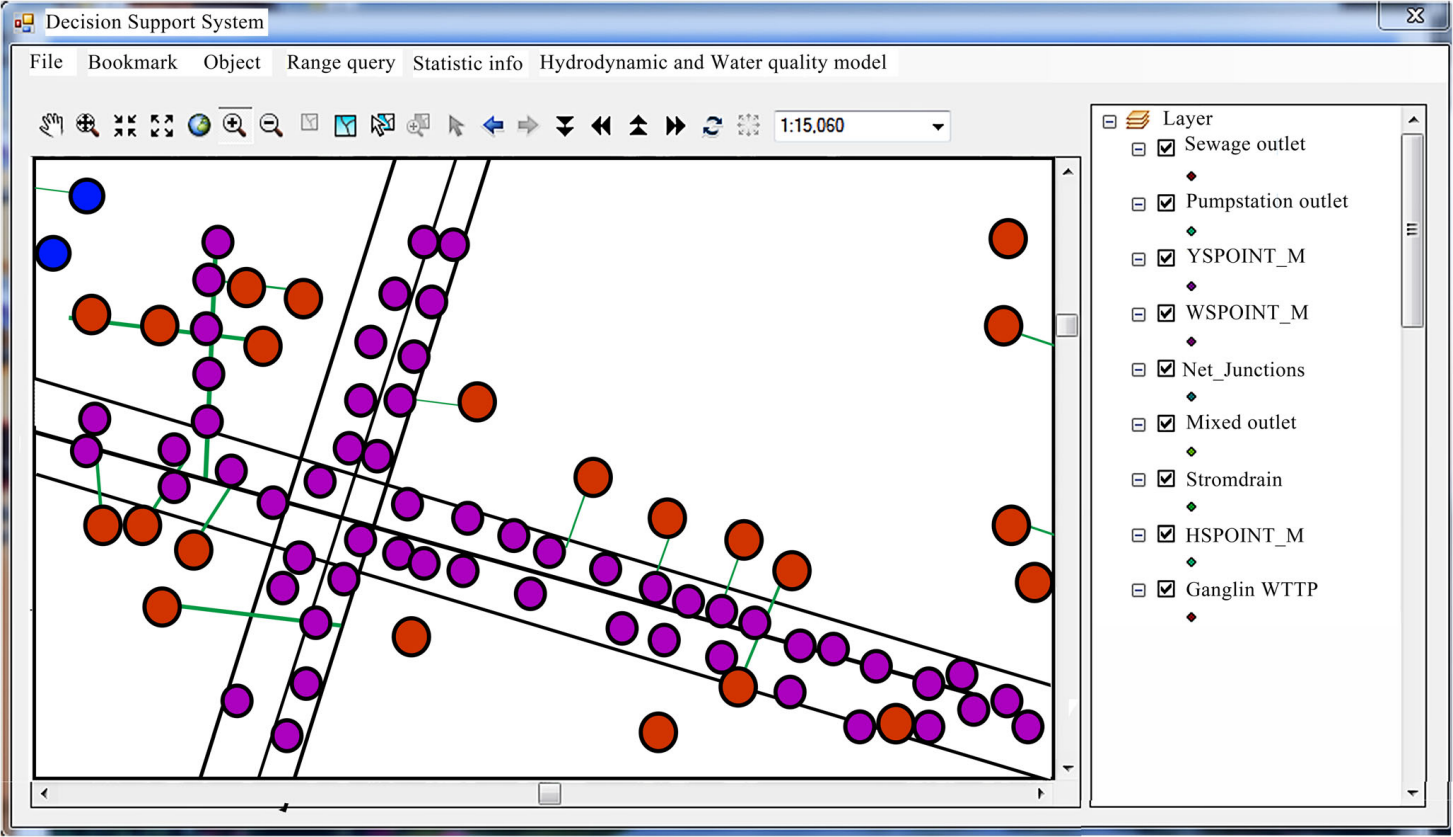
The urban water environment GIS platform

On the GIS map, the inland river system was generalized into seven rivers with 161 water level calculation points, 149 flow calculation points, and 310 water quality calculation points, totaling 310 sections. The urban water environment GIS platform in this study area is shown in Fig. 4.

### Display of application and results

**Fig. 5:**
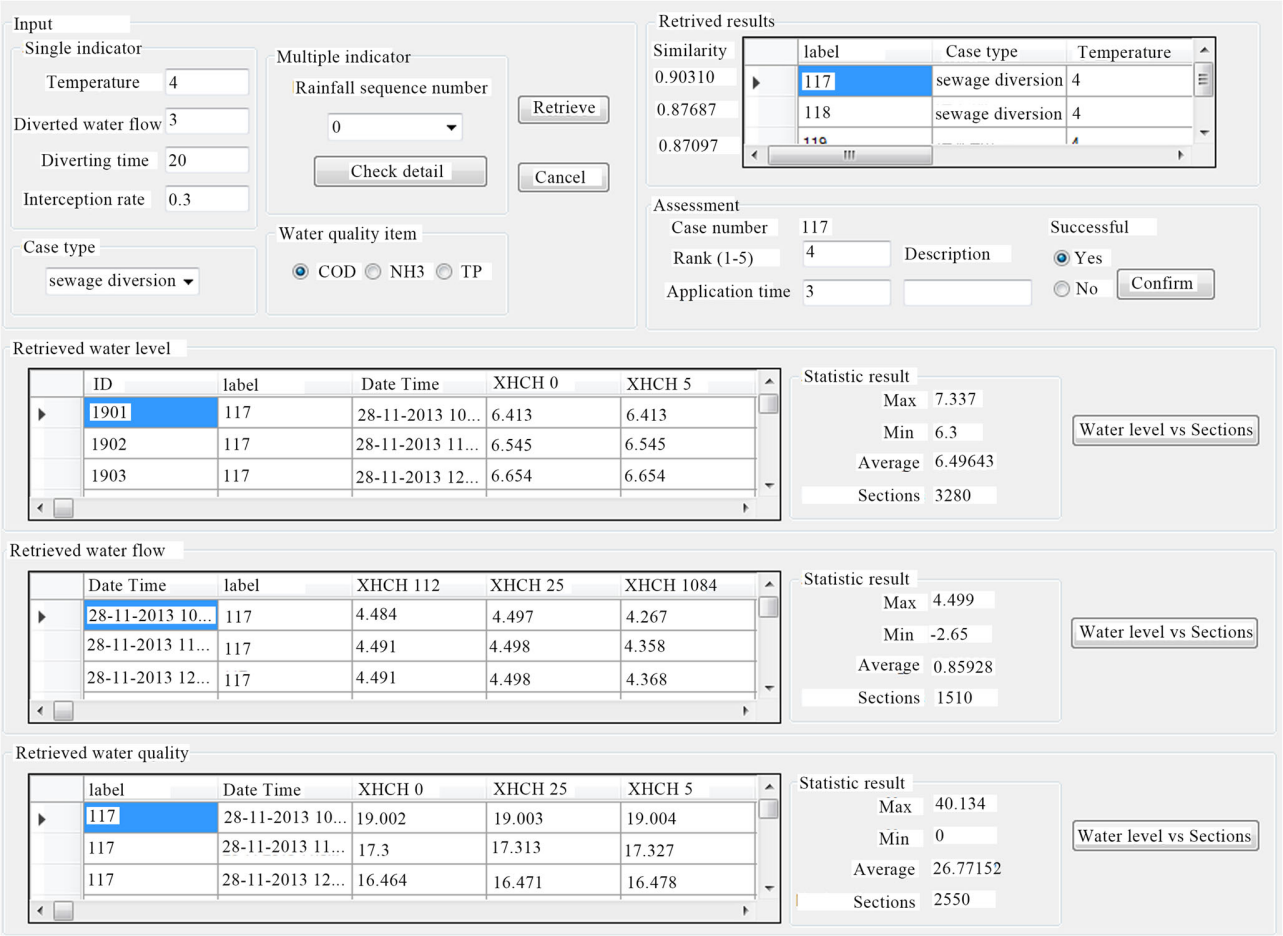
Interface of case retrieval

**Fig. 6:**
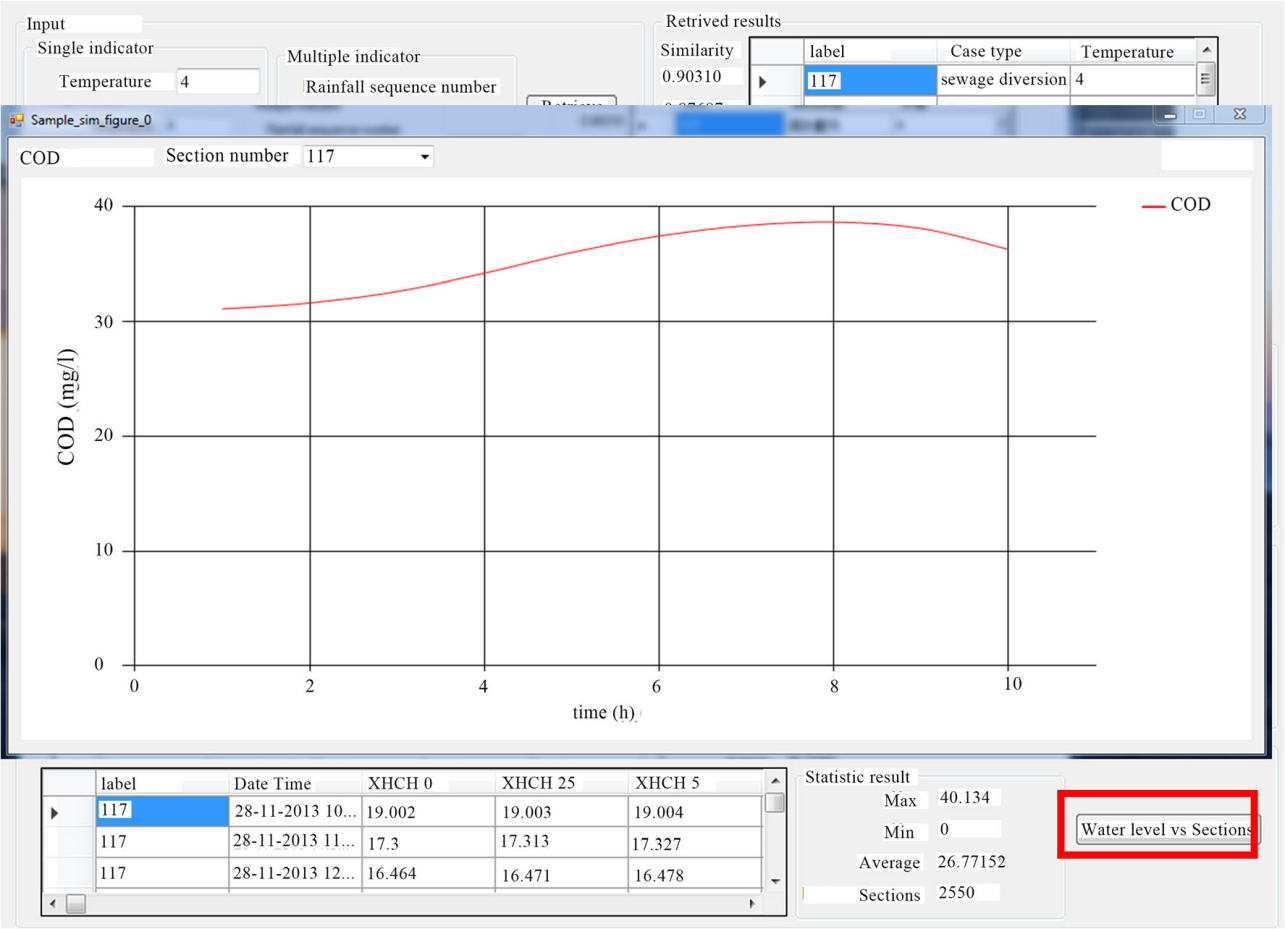
Curve of COD in the selected cross section

Assume that both water diversion and sewage interception were needed to be implemented in this study area. Upon starting the system, input the parameters (Fig. 5) on the interface of case retrieve and then click “retrieve,” you will quickly retrieve all the matching results called from the case base. According to the rule of similarity, the most similar case can be selected and the system will automatically display its hydrodynamic and water quality results. On the other hand, MSchart can also be used to generate the water level, water flow, and water quality profiles of the section. For example, when the most similar case numbered 117 and the water quality item were chosen, the curve of COD was illustrated (Fig. 6) in this section which shows COD decreased from 38 to 36 mg/L in 2 h. After the retrieved result successfully being applied, text can be entered to further evaluate the retrieval effectiveness.

As the traditional method goes, the operator need to model first when encountering a new problem and then manually imports results of the model calculation into GIS. In contrast, the CBR method is faster which avoids tedious modeling steps, greatly reducing the manpower and financial resources required and also facilitating the manager who may lack professional knowledge.

**Fig. 7:**
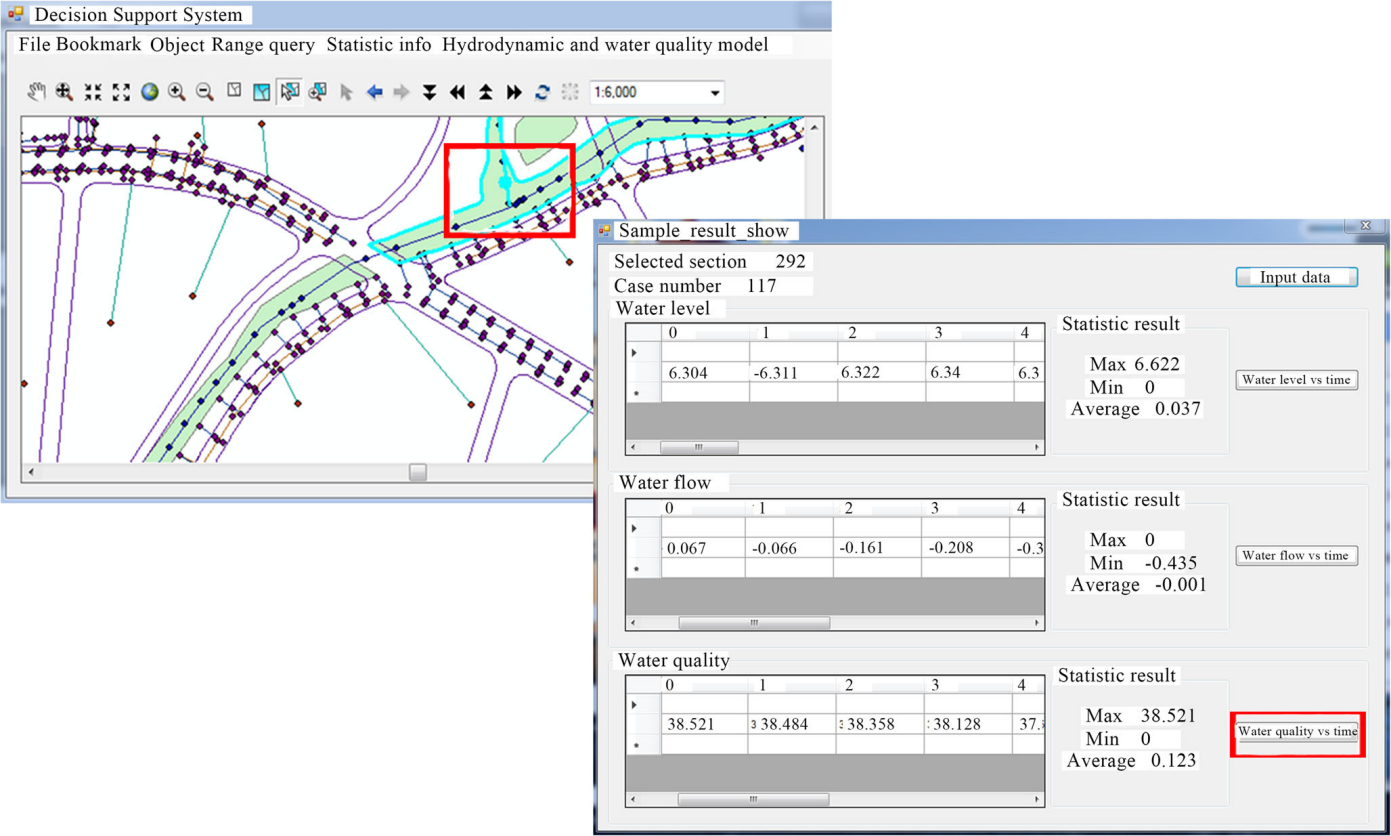
Interface of the selected cross section in GIS

**Fig. 8:**
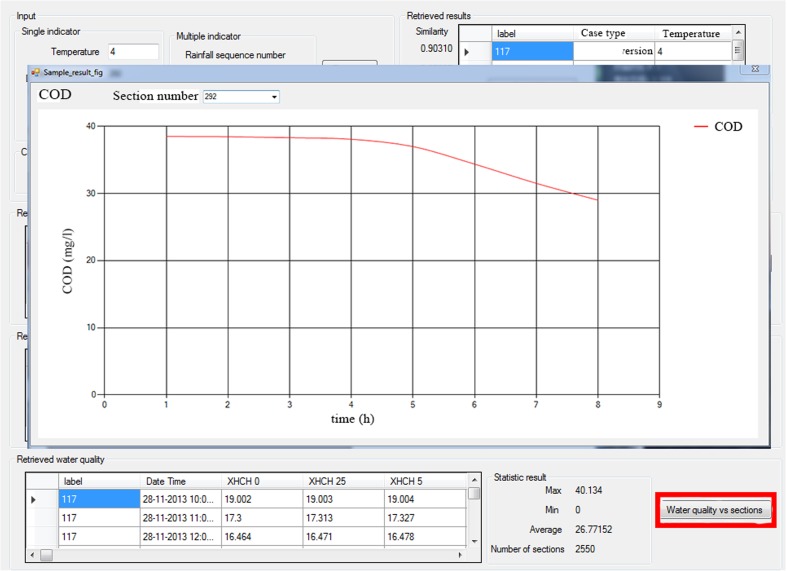
Curve of COD in the selected cross section in GIS

In addition to the above method of viewing the retrieved results, the operator can also query the hydrodynamic and water quality indicators of the specific section through its location on the map of GIS. That is, only a specific section selected on the map is required, the water quality and hydrodynamic time series data of the section can be directly obtained by using the CBR method (Fig. 7 and Fig. 8).

## Discussion

The paper presents a CBR-based integration of a hydrodynamic and water quality model and a GIS. Firstly, assume that there is not such a decision support system in Chaohu City. When encountering water environmental problems, it is necessary to invest great human and financial resources to let professional technicians carry out the tedious and complicated modeling calculation and analysis and then manually input the results into the GIS, which requires a long modeling and calculation processing time. Decision makers who lack of professional knowledge are difficult to understand and perform. In contrast, the CBR-based integration greatly simplifies the operation process and reduces the difficulty. Only a basic knowledge of GIS is required, the manager can invoke and visualize the case results in the GIS platform to facilitate his or her management decisions. At the same time, the economic investment of small- and medium-sized cities in China is also within their tolerance.

Secondly, CBR method proposed in this paper mainly has three advantages: fast, easy to operate, and economical.

**Fig. 9:**
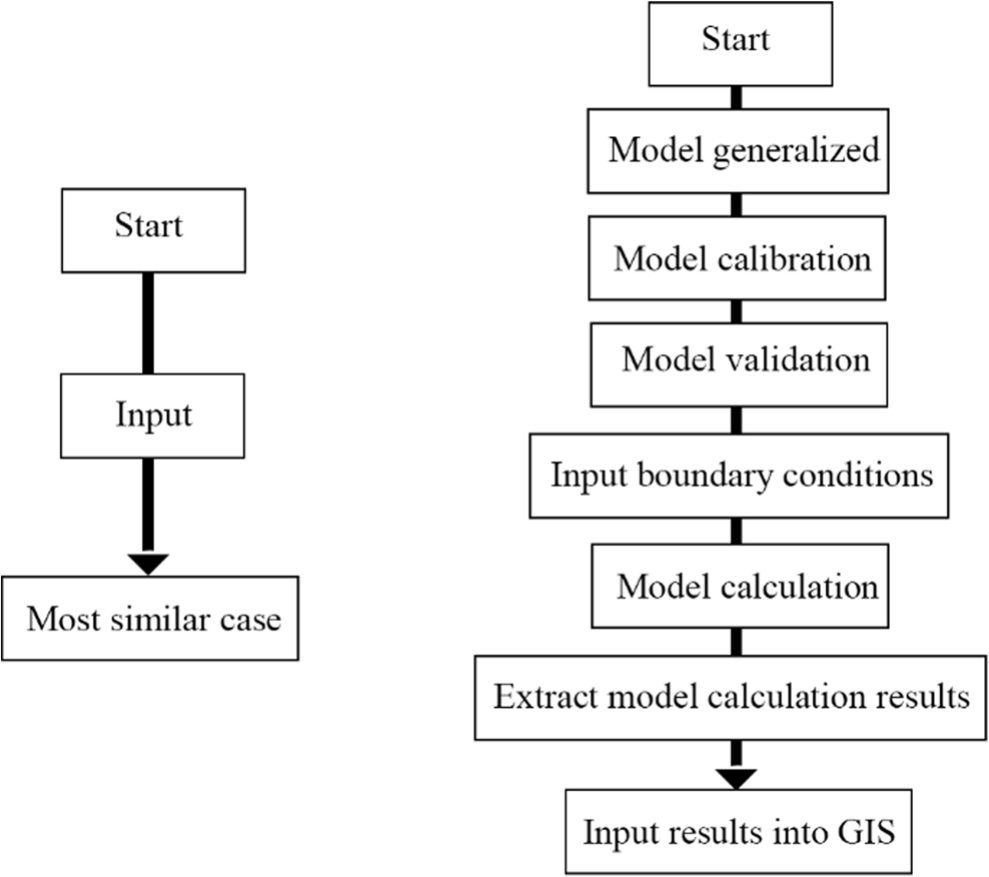
Instruction flowchart of CBR and MIKE11

1. Fast: Time needed to solve the same case by CBR and the model was compared. Calculation time consumed by CBR and MIKE11 was 6 s and 200 s, respectively, using the same PC with CPU of Intel i7-4700. It was obvious that based on CBR method, tedious model calculation of each new case can be avoided and the decision time of the manager can be greatly shortened.
2. Easy to operate: For managers, instruction flowchart of CBR and MIKE11 presented in this paper is shown in Fig. 9. It can be seen that the CBR method not only shortens decision time but also lowers the threshold for the usage of managers. New problems can be solved through a convenient operation interface without professional modeling foundation.
3. Economical: With the help of loose coupling approach and modular designing, the system achieves a simpler structure and easier maintaining routine. Accordingly, decomposable systems with a high degree of independence (loose coupling) were modular (Sanchez and Mahoney 1996). And modularity can reduce product cost as additional cost will be saved by simultaneously implementing strategies aimed at integrating functions and activities in design, manufacturing, or supply management (Jacobs et al. 2007). Also, it may be a good system for maintenance and localized adaptation. Single element in the system can modify a local unique contingency without affecting the whole system, which leads to a relatively economical and substantial local adaptation (Weick 1976).

Finally, however, this study needs to be further explored in the following two aspects:

1. At present, the CBR system basically relies on the embedded knowledge to revise the case. Under this condition, specific rules have to be formulated in advance. However, CBR is often used in areas with a poor understanding or it is difficult to code the rules. There is no uniform revise rule and the commonly used proportional revise method is just a simple revise of numerical attributes, which is hard to meet the requirements of complex situations. Solution of basic equations of the model should be more focused and changing rules of the quantitative data ought to be found to revise the case.
2. Case base needs to be expanded. The integrated system developed in this study is only a prototype system. The number of cases in the case base is relatively insufficient. Therefore, models should be used to simulate improvement of water environment under different engineering conditions and changes of river water quality under various external influences, adding various kinds of cases and inputting into the case base.

## Conclusion

The study has explored to integrate a hydrodynamic and water quality model and GIS based on the loose-coupling method-CBR. Under the premise of insufficient previous studies, it is innovatively proposed to use the model to simulate and save cases so as to build the case base, which realizes the integration of the hydrodynamic and water quality model and GIS by CBR. The functionality was illustrated through a case study of Chaohu City, which included case retrieval, results interpretation and the visual display in GIS platform. The advantages of the fast, easy-to-operate, economic integration can be summarized as follows: (1) the fused capability of water quality predication of models and strong spatial data processing analysis of GIS can be realized at the same time by integration; (2) results can be invoked from the case base by CBR, which overcomes tedious modeling steps and a large number of model calculations needed before and thus reduces the time needed to understand the usage of the model especially for first-time users; (3) the user-friendly interface makes it more convenient for decision makers to use the model efficiently and quickly, which improves the application of the model.

## Appendix

Detailed information of the balanced equation of DO, COD, TP, and NH_3_-N were shown as follows:

5 $$ \frac{dDO}{dT}={K}_2\bullet \left({C}_s- DO\right)-{R}_{20}\bullet {\theta}_2^{\left(T-20\right)}+P(t)-{K}_3\bullet COD\bullet {\theta}_3^{\left(T-20\right)}-{B}_1\bullet {\theta}_1^{\left(T-20\right)}-{Y}_1\bullet {K}_4\bullet {NH}_3\bullet {\theta}_4^{\left(T-20\right)} $$

6 $$ \frac{dCOD}{dT}=-{K}_3\bullet COD\bullet {\theta}_3^{\left(T-20\right)}-\frac{K_5}{depth}\bullet COD+\frac{S_1}{depth}\bullet COD $$

7 $$ \frac{ dT P}{dT}=+{Y}_3\bullet {K}_3\bullet COD\bullet {\theta}_3^{\left(T-20\right)}-\left(P-R\right)\bullet {U}_3-\frac{K_6}{depth}\bullet TP+\frac{S_2}{depth}\bullet TP $$

8 $$ \frac{d{NH}_3}{dT}=+{Y}_2\bullet {K}_3\bullet COD\bullet {\theta}_3^{\left(T-20\right)}-{K}_4\bullet {NH}_3\bullet {\theta}_4^{\left(T-20\right)}-\left(P-R\right)\bullet {U}_1-{U}_2\bullet {K}_3\bullet COD\bullet {\theta}_3^{\left(T-20\right)} $$

The values of the parameters are shown in Table 8.

**Table 8:** Detailed information of parameters in the above formulas

Parameters	Descriptions	Unit
*K*_2_	Reaeration coefficient at 20 °C	/day
*DO*	The dissolved oxygen concentration of water	mg/L
*C*_*s*_	Saturation values of dissolved oxygen	mg/L
*R*_20_	Respiration rate of animals and plants	g O_2_/(m^3^ day)
*T*	Water temperature	°C
*P*_*(t)*_	Photosynthesis coefficient associated with time	G O_2_/(m^2^ day)
*K*_*3*_	Degradation coefficient of COD at 20 °C	/day
*θ*_1_, *θ*_2_, *θ*_3_, *θ*_4_	Temperature coefficient for SOD, respiratory processes, COD degradation, nitrification	Dimensionless
*B*_1_	Sediment oxygen demand at 20 °C	/day
*COD*	The COD concentration of water	mg/L
*NH*_3_	The NH_3_-N concentration of water	mg/L
*K*_4_	The nitrification rate at 20 °C	/day
*Y*_1_	Oxygen demand by nitrification	G O_2_/g NH_3_-N
*K*_5_	Sedimentation rate of COD	m/day
*S*_1_	Resuspension rate of COD	m/day
Depth	Depth of water	M
*Y*_3_	Ratio of phosphorus released at COD decay	g P/g COD
*U*_3_	Uptake of P in plants by photosynthesis	g P/g O_2_
*K*_6_	Sedimentation rate of TP	m/day
*S*_1_	Resuspension rate of TP	m/day
*Y*_2_	Ratio of ammonia released at COD decay	g NH_3_-N/g COD
*U*_1_	Uptake of ammonia in plants by photosynthesis	g NH_3_-N/g O_2_
*U*_3_	Uptake of ammonia in bacteria	g NH_3_-N/g O_2_
